# Mycobacterial infections in wild boars (*Sus scrofa*) from Southern Switzerland: Diagnostic improvements, epidemiological situation and zoonotic potential

**DOI:** 10.1111/tbed.13717

**Published:** 2020-07-20

**Authors:** Giovanni Ghielmetti, Monika Hilbe, Ute Friedel, Chiara Menegatti, Luca Bacciarini, Roger Stephan, Guido Bloemberg

**Affiliations:** ^1^ Institute for Food Safety and Hygiene Section of Veterinary Bacteriology University of Zurich Zurich Switzerland; ^2^ Institute of Veterinary Pathology University of Zurich Zurich Switzerland; ^3^ Cantonal Veterinary Office Bellinzona Switzerland; ^4^ Institute for Food Safety and Hygiene National Reference Center for Enteropathogenic Bacteria and Listeria University of Zurich Zurich Switzerland

**Keywords:** 16S rRNA gene, *hsp65*, MALDI‐TOF MS, *Mycobacterium avium*, *Mycobacterium microti*, non‐tuberculous mycobacteria, *rpoB*, wild boar

## Abstract

The occurrence of mycobacterial infections in different hosts and their implication as obligate or opportunistic pathogens remain mainly unclear. In addition to the well‐known pathogenic members of the *Mycobacterium tuberculosis* – complex (MTBC), over 180 non‐tuberculous mycobacteria (NTM) species have been described. Although the large majority of the NTM is assumed to be non‐pathogenic to most individuals, an increasing trend in NTM infections has been observed over the last decades. The reasons of such augmentation are probably more than one: improved laboratory diagnostics, an increasing number of immunocompromised patients and individuals with lung damage are some of the possible aspects. Mandibular lymph nodes of 176 hunted wild boars from the pre‐Alpine region of Canton Ticino, Switzerland, were collected. Following gross inspection, each lymph node was subjected to culture and to an IS*6110* based real‐time PCR specific for MTBC members. Histology was performed of a selection of lymph nodes (*n* = 14) presenting gross visible lesions. Moreover, accuracy of matrix‐assisted laser desorption ionization time‐of‐flight mass spectrometry (MALDI‐TOF MS) species identification was compared with sequence analysis of a combination of housekeeping genes. Mycobacteria of the MTBC were detected in 2.8% of the wild boars (*n* = 5; CI_95%_ 1.2–6.5) and were all confirmed to be *Mycobacterium microti* by molecular methods. In addition, based on the examined lymph nodes, NTM were detected in 57.4% (*n* = 101; CI_95%_ 50.0–64.5) of the wild boars originating from the study area. The 111 isolates belonged to 24 known species and three potentially undescribed *Mycobacterium* species. *M. avium* subsp. *hominissuis* thereby predominated (22.5%) and was found in lymph nodes with and without macroscopic changes. Overall, the present findings show that, with the exception of undescribed *Mycobacterium* species where identification was not possible (3.6%; 4/111), MALDI‐TOF MS had a high concordance rate (90.1%; 100/111 isolates) to the sequence‐based reference method.

## 1 INTRODUCTION

Bovine tuberculosis (bTB) is a chronic disease caused by members of the *Mycobacterium tuberculosis* complex (MTBC; Rodriguez‐Campos, Smith, Boniotti, & Aranaz, 2014). MTBC have been isolated from numerous different domestic and wild animal species. Recent epidemiological investigations have shown the fundamental role played by wildlife in the maintenance of the causal agents of bTB. This results in continuous interspecies transmissions from wild animals to livestock and vice versa, hindering national and international eradication programs (Atkins & Robinson, 2013; Fink et al., 2015; Garcia‐Jimenez et al., 2016; Nigsch, Glawischnig, Bago, & Greber, 2018). Badger (*Meles meles*), free‐ranging red deer (*Cervus elaphus elaphus*) and wild boar (*Sus scrofa*) are the most relevant known wild animals acting as a reservoir of bTB in Europe. The ongoing geographic expansion of wild boar populations has raised concerns regarding the monitoring of several infectious diseases, including zoonotic plagues like bTB and hepatitis E (Gortazar et al., 2008; Martinelli et al., 2015). Beside the members of the MTBC and *Mycobacterium leprae*, the agent that causes Hansen's disease, over 180 species of non‐tuberculous mycobacteria (NTM) have been described (Gupta, Lo, & Son, 2018). NTM are commonly encountered in the environment and they have been isolated from a variety of sources, including water, feed, soil, dust, aerosol, protozoa and animals (Falkinham, 2015; Ghielmetti et al., 2018). Of these, two species are recognized as true pathogens for humans, namely *M. marinum* and *M. ulcerans* (Johansen, Herrmann, & Kremer, 2020). Nevertheless, more than 60 species of NTM are known to be opportunistic pathogenic to humans and other mammals, and infections with these emerging pathogens are now more common than tuberculosis in industrialized countries (Biet & Boschiroli, 2014; Griffith et al., 2007; Tortoli, 2014). Immunocompromised individuals are highly susceptible to opportunistic NTM infections and improved laboratory diagnostics have enabled more accurate detection of fastidious or extremely slow growing species. Despite the increasing relevance of mycobacterial infections, only restricted information on the occurrence and their diversity in wildlife is available. Moreover, although wild boars are among the most widely distributed large mammals in the world (Oliver, IUCN/SSC Pigs, and Peccaries Specialist Group, & IUCN/SSC Hippo Specialist Group, 1993), the literature concerning this species is mainly focused on the presence of MTBC and the impact of NTM infections on the prevalence of MTBC (Boniotti et al., 2014; Chiari et al., 2016; Di Marco et al., 2012; Michelet et al., 2015; Naranjo, Gortazar, Vicente, & de la Fuente, 2008; Richomme, Boschiroli, Hars, Casabianca, & Ducrot, 2010; Santos et al., 2009; Vicente et al., 2006). Canton Ticino is the most southern Canton of Switzerland, and a large proportion of its borders is shared with Italy. The territory encompasses an area of 2,812 km^2^, where the majority of the urban area is concentrated in the flat land and forests cover about one third of the alpine region. The wild boar presence in the territory has been documented during the XVI century. Thereafter, it disappeared and it is only since 1981 that it has been officially sighted again (Dipartimento Territorio/ Finanze e Economia, 2010). The wild boars are distributed almost exclusively along flat and hilly land, with the highest animal density observed in the southern districts of Mendrisio, Lugano and the lower part of the Maggia Valley (Dipartimento Territorio/ Finanze e Economia, 2010). The population increase is estimated at 100%–180%; consequently, the population can theoretically double or even triplicate in 12 months without control measurements. In order to regulate this growth, licensed hunters are allowed to hunt the animals each year in September without sex or hunting bag restrictions. Wild boar is one of the most hunted mammals in the Canton of Ticino, second only to red deer.

Recent studies from Spain, Czech Republic, Brazil and Slovenia comprehensively evaluated the spectrum of NTM species in black pigs using molecular methods (Garcia‐Jimenez et al., 2015; Gortazar et al., 2011; Munoz‐Mendoza et al., 2013; Pate et al., 2016; Trcka et al., 2006). It is significant to note that, the latter mentioned publications, describe marked differences in the spectrum of species isolated. Such differences may not be exclusively the result of geographic distribution of NTM. The advance in molecular techniques and the progress in mycobacterial characterization led to enormous diagnostic improvements over the past decades (Tortoli, 2014). It is noteworthy that the dissection of the *M. avium* complex (MAC) in the mentioned publications was performed at different levels, impeding a direct comparison of the isolated mycobacteria. The characterization of NTM from clinical samples is often a challenge for laboratory personal in routine diagnostic. Because of its rapidness, cost‐effectiveness, and high throughput, the matrix‐assisted laser desorption ionization time‐of‐flight mass spectrometry (MALDI‐TOF MS) technology has nowadays been integrated in the workflow of numerous diagnostic laboratories (Alcaide et al., 2018; Mediavilla‐Gradolph et al., 2015; Murugaiyan et al., 2018). However, the accuracy achievable at present with genetic approaches remains superior to the MALDI‐TOF MS‐based species identification (Tortoli, 2014). Therefore, the accuracy and limitations of this method based on ordinary samples from veterinary origin should be evaluated. The present research used a panel of NTM showing a wide range of species isolated from a common source and verified the consensus grade between sequence analysis and MALDI‐TOF MS. This study aimed (a) to determine the occurrence and diversity of mycobacterial species among healthy wild boar hunted in the Canton of Ticino, (b) to identify the geographical distribution of mycobacterial species and (c) to compare two different diagnostic identification approaches for the genus *Mycobacterium*.

## 2 MATERIALS AND METHODS

### 2.1 Collection of samples

Hunting seasons in Switzerland are regulated independently by the Cantonal Veterinary Offices. In total, 1,436 and 1,588 wild boars were hunted in the Canton of Ticino in 2017 and 2018, respectively. Mandibular lymph nodes of 86 and 90 animals were collected during the two hunting periods of the study and the weight of every sampled wild boar was determined. Assuming an estimated population size of 2,500–3,000 animals, approximatively 3% of the population was analysed for two subsequent years.

Sex, age and the exact geographical position of each animal were recorded directly on the field. Age classification was based on tooth eruption patterns: animals <6 months of age were recorded as juveniles, those between 6 months and 2 years of age as yearlings, and adult animals older than 2 years composed the last group. Trained personnel of the Cantonal Veterinary Officeexcised lymph nodes by using sterile dissection knives and scalpels. Each sample was immediately packed into sterile containers and transported to the laboratory under cooled conditions.

### 2.2 Microbiological procedures

Samples preparation and mycobacterial cultures were performed as described elsewhere (Ghielmetti et al., 2018). Briefly, BBL MGIT liquid media tubes supplemented with Bactec MGIT 960 growth supplement, BBL MGIT PANTA (Polymyxin B, Amphotericin B, Nalidixic acid, Trimethoprim, Azlocillin) antibiotic mixture (Becton, Dickinson, BD) and 50 μg/ml sodium‐pyruvate were each inoculated with 0.5 ml of decontaminated and homogenized specimen. In addition, one Löwenstein‐Jensen and one Stonebrink agar slants (BD) were inoculated with the same inoculum and incubated up to 8 weeks at 37°C. In order to obtain pure mycobacterial cultures, subcultures on 7H10 agar‐plates and on Stonebrink agar slants (BD) were performed at intervals of three to ten days. Simultaneously, 400 μl of culture inoculum was suspended in 100 μl ATL buffer (Qiagen) and transferred onto a Lysing Matrix E tubes (MP Biomedicals). Genomic DNA was extracted through mechanical cell lysis using a TissueLyser II (Qiagen) and enzymatic digestion with Proteinase K (Qiagen) overnight. Automated DNA purification was performed using the QIAcube instrument in accordance with the QIAamp cador Pathogen Mini Kit protocol (Qiagen). DNA concentration in the final eluate was measured by reading the absorbance at 260 nm using a NanoDrop 2000c Spectrophotometer (Thermo Fisher Scientific), diluted to a maximal concentration of 100 ng/µl and stored at −20°C until use. Purified DNA was used for direct MTBC detection using the qPCR assay targeting insertion sequence *IS6110* as described by Reed et al. with slight modifications (Reed et al., 2016). Briefly, the MTB IS6110 probe was double‐quenched with iQ500 and BHQ1 instead of ZEN and 3IABkFQ, respectively. Moreover, the qPCR assay internal control was substituted by eGFP as described by Hoffmann et al. and performed on a 7500 Fast real‐time PCR system (Applied Biosystems; Hoffmann, Depner, Schirrmeier, & Beer, 2006).

DNA from cultured mycobacteria was extracted by inoculating a loop‐full of cell materials into 200 μl of chelating ion‐exchange resin (InstaGene Matrix) and centrifuged at 13,000 *g* for 10 min. The supernatant was used in downstream reactions.

Pure cultures that presented acid‐fast bacilli (AFBs) by Ziehl‐Neelsen (ZN) staining and negative MTBC qPCR results were classified as NTM and further characterized by sequence analysis of a combination of housekeeping genes and matrix‐assisted laser desorption/ionization time‐of‐flight mass spectrometry (MALDI‐TOF MS). Sanger sequencing of 16S rRNA (Scherrer, Landolt, Carroli, & Stephan, 2018), *rpoB* (Adékambi, Colson, & Drancourt, 2003) and *hsp65* (Telenti et al., 1993) housekeeping genes was performed in duplicates followed by gene homology analyses. For isolates identified as members of the MAC, the complete *hsp65* gene was sequenced as proposed by Turenne et al. using primers MAChsp65F and MAChsp65R (Turenne, Semret, Cousins, Collins, & Behr, 2006). DNA sequencing was performed at Microsynth. Resulting sequences were assembled using CLC Genomics Workbench 7.5.1 (Qiagen) and BLAST similarity searching for multiple sequence alignment was performed (https://blast.ncbi.nlm.nih.gov/Blast.cgi). Control strains included were *M. avium* subsp*. avium* ATCC 25291, *M. avium* subsp*. hominissuis* ATCC 700898 and *M. peregrinum* ATCC 700686. For samples resulted positive by direct MTBC real‐time PCR, mycobacteria could not be cultivated from any of the described culture media, even after 12 months of incubation. Molecular characterization by mycobacterial interspersed repetitive unit and variable number tandem repeats (MIRU‐VNTR) and species determination using spoligotyping were therefore performed using DNA extracts of lymph nodes as previously described (Ghielmetti et al., 2017).

### 2.3 MALDI‐TOF mass spectrometry

Inactivation and preparation of the isolates for MALDI‐TOF MS analysis was performed using the Mycobacteria Extraction Method (MycoEX) in accordance with the manufacturer. In order to enable optical evaluation of the tested colonies and because the quality of the spectra obtained from isolates grown on solid media is better than those obtained from liquid media, 7H10 agar plates were chosen as culture medium for MALDI‐TOF MS analysis (Kodana et al., 2016; Lotz et al., 2010). A loopful of culture from solid medium was transferred into a 1.5 ml Eppendorf tube with 300 μl of HPLC‐water and inactivated for 30 min at 99°C under biosafety level 3 conditions. After a centrifugation step of 2 min at 13,000 *g*, the supernatant was discharged the pellet was re‐suspended in 300 μl of HPLC‐water and 900 μl of ethanol. Thereafter, centrifugation was repeated, and the supernatant was discharged. The tubes were left open enabling the pellets to dry at room temperature. A spatula‐tip full of bead suspension (Zirconia/Silica; BioSpec) and 10–50 μl of acetonitrile were added to the pellets, depending on the volume of the pellets. Mycobacterial cells were disrupted by vortexing at maximal speed for 1 min and 25–50 μl of 70% formic acid were added, depending on the volume of the pellets. In conclusion, the tubes were centrifuged at the same conditions as above and 1 μl of the supernatant was spotted on the MALDI‐TOF target plates (MSP 96 target ground steel; Bruker Daltonics) in duplicates. At this point, the target plates were allowed to dry at room temperature and then taken to biosafety level 2 conditions. Thereafter, 1 μl of matrix was added to each spot (HCCA, α‐cyano‐4‐hydroxycinnamic acid).

Peptide mass spectra were acquired in a linear positive ion mode at a maximum laser frequency of 60 Hz across a mass to charge ratio (m/z) of 2,000 to 20,000 Da using the Microflex LT benchtop operating system (Bruker Daltonik GmbH). Each spot was measured twice using the MBT_FC.par FlexControl method and analysed by the FlexAnalysis 3.3 software (Bruker Daltonik GmbH). The highest log score value was compared with the MBT Mycobacteria Library 4.0, containing 880 main spectrum profiles (MSP), representing 159 mycobacterial species. An *Escherichia coli* reference strain provided by the manufacturer was used in each run as a calibrator and for quality control. Log scores values (LSV) between 2.0 and 3.0 were considered as acceptable and between 1.8 and 2.0 were treated with caution and considered consistent when the same species was the only one suggested by the software with a LSV above 1.8. Lower LSV (<1.8) were interpreted as incorrect and recorded as 'no identification possible' (Saleeb, Drake, Murray, & Zelazny, 2011). For each species isolated, the measured spectra of one isolate were exported in specific main spectrum profiles (MSP) using the MALDI Biotyper Compass Explorer 4.1 software. Each MSP was matched against the MBT Mycobacteria Library 4.0.

### 2.4 Macroscopic and histological examination

After removal of fat and connective tissue, qualified staff inspected the lymph nodes macroscopically and recorded pathological changes. A subset of samples was additionally submitted for histology (*n* = 14). Selection criteria were focusing on infections with the seven most prevalent *Mycobacterium* species and presenting macroscopic lesions. In addition, all lymph nodes that tested positive for MTBC by real‐time PCR were submitted for histological examination (*n* = 5). Samples were fixed in 10% buffered formalin and embedded in paraffin. Two to three‐micron‐thick tissue sections were obtained and stained with haematoxylin and eosin (HE). Additionally, in cases where lesions consistent with mycobacterial infections were observed after HE staining, an additional ZN staining was performed.

#### 2.4.1 Geographical distribution and statistical analysis

The geographical distribution of lymph nodes showing growth of mycobacteria and the circulation of the different NTM on the territory were investigated using the free software QGIS Desktop 3.6.1. Based on the collected data regarding sex and age of the animals, statistical analysis was performed using GraphPad Prism 8.2.1 (GraphPad Software). Fisher's exact test was used to evaluate different age groups and the presence of viable NTM isolated from their lymph nodes. Moreover, a possible association between the isolation of MAC and the three age groups was investigated with the same test. Statistical significance was set to *p* < .05.

### 2.5 Ethics statement

All animal samples used in this study originated from legally hunted wild boars in accordance with the Swiss legislation (Hunting Law SR 922 and Animal Welfare Act SR 455). An ethical approval or permit for animal experimentation was not applicable.

## 3 RESULTS

A total of 176 wild boars were collected during 2017 and 2018 of which 40.3% were juveniles (*n* = 71), 33.5% yearlings (*n* = 59), 24.4% adults (*n* = 43) and 1.7% undetermined (*n* = 3). The mandibular lymph nodes of 101 (57.4%; CI_95%_ 50.0–64.5) animals showed typical growth of *Mycobacterium* spp. (111 isolates in total) and AFBs by ZN staining were observed. The sequenced genes (16S rRNA, *rpoB*, *hsp65*) assigned 108 of 111 isolates to 24 described *Mycobacterium* species (Table 1). Three single isolates could not be classified to any known mycobacterial species and may represent new species. *M. avium* subsp. *hominissuis* (*Mah*) predominated with 22.5% of the isolates, followed by *M. nonchromogenicum* with 21.6% of the isolates.

**TABLE 1:** Identification of non‐tuberculous mycobacteria isolated from wild boars and human clinical relevance. A total of 111 non‐tuberculous mycobacteria isolates belonging to 24 known species and three potentially undescribed *Mycobacterium* species were cultured from 176 wild boar mandibular lymph nodes. Sequence analysis was used as gold standard method. Results of identifications based on MALDI‐TOF MS applying two different log scores value cut‐offs are shown. Discrepant results intended as (a) no species identification by MALDI‐TOF analysis or (b) assignment of a discrepant species compared to sequence analysis are highlighted

Species identified by sequence analysis (16S rRNA, rpoB and hsp65)	No. of isolates	Identification by MALDI‐TOF MS		Human clinical relevance^a^
		LSV > 2	LSV > 1.8	
*Mycobacterium avium*	25	24	25	+ (van Ingen et al., 2018)
*Mycobacterium nonchromogenicum*	24	15	24	+ (Sawai et al., 2006)
*Mycobacterium vaccae*	8	8	8	‐
*Mycobacterium engbaekii*	6	3	6	‐
*Mycobacterium neoaurum*	6	4	6	+ (Walayat, Awwal, Roy, & Ahmad, 2018)
*Mycobacterium nebraskense*	4	1	4	+ (Tortoli, 2006)
*Mycobacterium lentiflavum*	4	4	4	+ (Yagi et al., 2018)
*Mycobacterium colombiense*	3	0	0	+ (Tortoli, 2006)
*Mycobacterium diernhoferi*	3	3	3	‐
*Mycobacterium florentinum*	3	2	3	+ (Tortoli et al., 2005)
*Mycobacterium peregrinum*	3	3	3	+ (Hoefsloot et al., 2013)
*Mycobacterium bourgelatii*	2	2	2	‐
*Mycobacterium celatum*	2	2	2	+ (Chavarria, Lutwick, & Dickinson, 2018)
*Mycobacterium chimaera intracellulare* group	2	2	2	+ (van Ingen et al., 2018)
*Mycobacterium intermedium*	2	2	2	‐
*Mycobacterium scrofulaceum*	2	0	0	+ (Wilson, Jagtiani, & Wengenack, 2019)
*Mycobacterium phlei*	2	1	2	+ (Tanaka et al., 2019)
*Mycobacterium fortuitum complex*	1	1	1	+ (Hoefsloot et al., 2013)
*Mycobacterium holsaticum*	1	1	1	+ (Richter et al., 2002)
*Mycobacterium interjectum*	1	1	1	+ (Dholakia, 2017)
*Mycobacterium monacense*	1	0	0	+ (Tortoli, 2014)
*Mycobacterium septicum*	1	1	1	+ (Schinsky et al., 2000)
*Mycobacterium terrae* complex	1	0	0	/
*Mycobacterium vulneris*	1	0	0	+ (Tortoli, 2014)
*Mycobacterium sp*. (not described)	3	0	0	/
Total	111	80	100	

^a^ Human clinical relevance according to previously reported cased (opportunistic pathogen: +; unknown pathogenicity or saprophytes: ‐).

**FIGURE 1:**
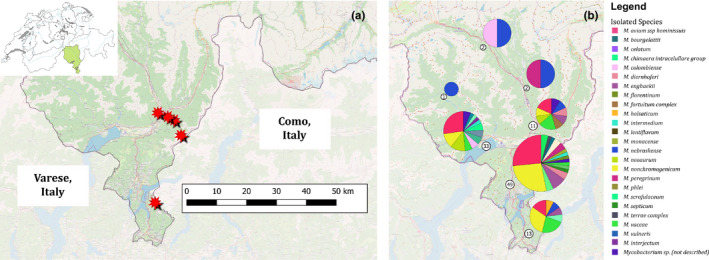
Geographic distribution of mycobacterial species identified from wild boars during 2017 and 2018 in the Canton of Ticino in Southern Switzerland (green highlighted in the country map of Switzerland in Panel (a). (a) Hunting location of the five animals infected with *Mycobacterium microti* (red stars) and (b) distribution of isolated non‐tuberculous mycobacterial species. Circled numbers represent the number of infected animals in each district

Five samples derived from three juvenile and two adult wild boars were positive by MTBC real‐time PCR. Since no growth of mycobacteria over a 12‐month incubation period could be achieved, species identification by direct spoligotyping using extracted DNA from the lymph nodes was performed. All five samples presented the same spoligotype signature SB0118, characterized by the presence of spacers 37–38 (www.Mbovis.org). The same signature is also known in the international spoligotyping database SpolDB4 as ST 539 and is indicative for *M. microti* (Brudey et al., 2006). Mycobacterial co‐infections were detected in nine samples (5.1%), originating from adults (*n* = 7) and juveniles (*n* = 2) and comprising one *M. microti* positive lymph node. Seven animals presented co‐infections with two NTM (*M. avium*/*M. vaccae*, twice *M. avium*/*M. nonchromogenicum*, *M. phlei*/*M. nonchromogenicum*, *M. nonchromogenicum*/*M. chimaera intracellulare* group, *M. scrofulaceum*/*M. florentinum*, *M. vaccae*/*Mycobacterium* sp.) one animal presented co‐infection with three NTM (*M. fortuitum* complex/*M. engbaekii*/*M. vaccae*) and one animal *M. microti*/*M. neoaurum*. Molecular characterization based on 24 MIRU‐VNTR loci was performed on four out of five *M. microti* containing samples and showed an identical code, suggesting a common source of infection or a transmission chain (Supplementary Material S1). The geographical localization of the wild boars infected with *M. microti* is displayed in Figure 1a.

**FIGURE 2:**
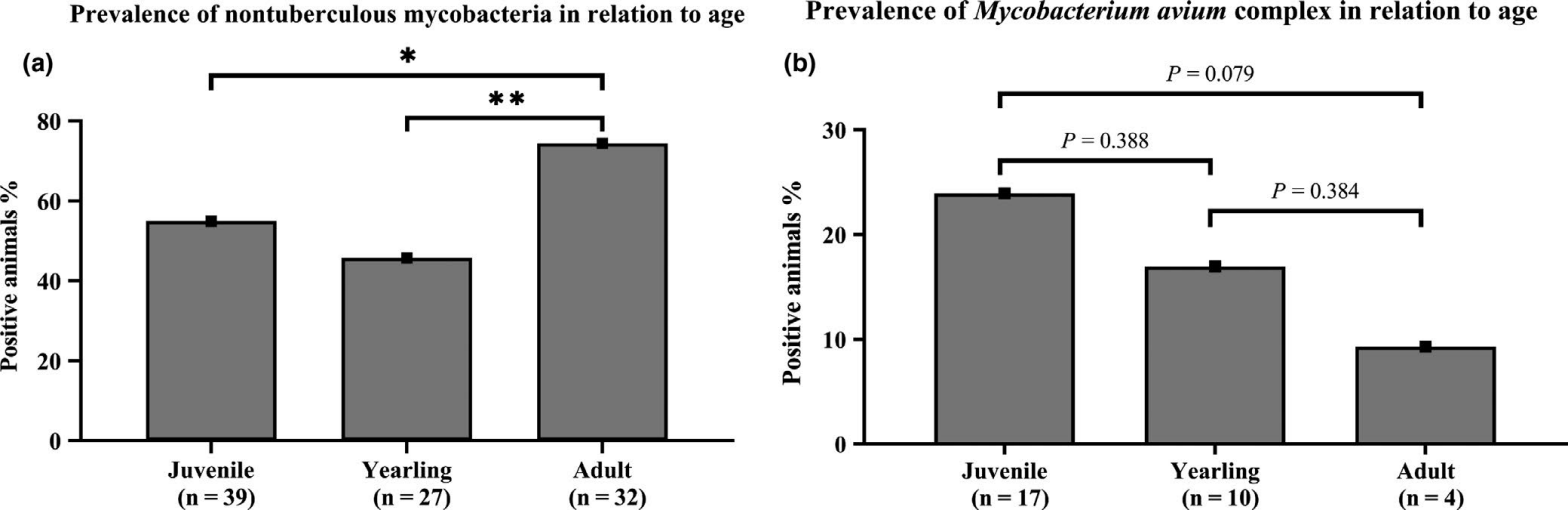
Prevalence of non‐tuberculous mycobacteria in relation to three different age groups (juvenile, yearling and adult). (a) Adult wild boars were more prone to be infected with non‐tuberculous mycobacteria (74.4%) in comparison with juvenile animals (54.9%) or yearlings (45.8%). No significant difference was observed between juvenile and yearlings (*p* = .3785). **, *p* ≤ .01; *, *p* ≤ .05 (compared to all other age groups and as indicated). (b) The prevalence of *Mycobacterium avium* complex in relation to age groups is shown. No statistically significant correlation between MAC and age of the animals was observed in the present survey

Adult wild boars were more prone to be infected with mycobacteria in comparison with juvenile animals or yearlings (Figure 2a). Overall, 74.4% of the analysed lymph nodes originating from adult animals showed growth of mycobacteria. Only 54.9% and 45.8% of the juveniles and yearlings presented viable NTM, respectively (*p* < .05). A correlation between infected animals and their sex was not found, nor a significant association between the isolation of MAC and the three age groups (juveniles vs yearlings *p* = .388; juveniles vs adults *p* = .079; yearlings vs adults *p* = .384; Figure 2b).

The distribution of the different NTM circulating among the analysed animals was investigated based on the geographical data collected. The hunted wild pig population was divided into seven districts of the study area and, with the exception of the three northern districts where a low number of samples were obtained; a homogeneous distribution of the NTM cultured is shown in the remaining four districts (Figure 1b).

### 3.1 Macroscopic and histological examination

**FIGURE 3:**
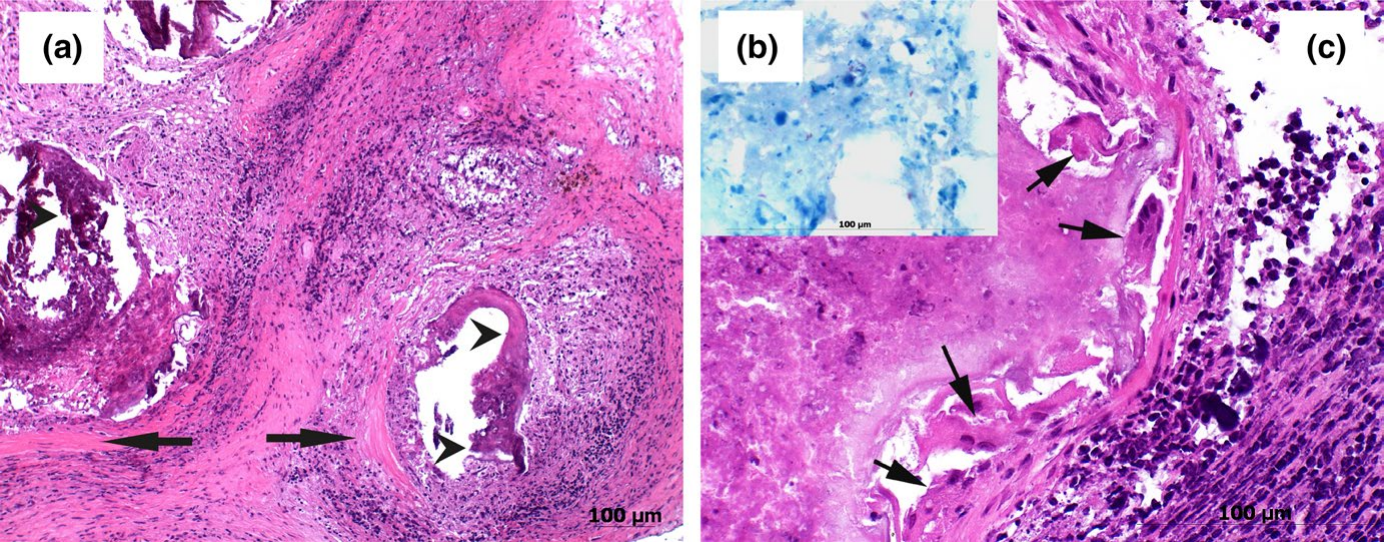
Histological findings from a mandibular lymph node presenting histological lesions compatible with tuberculosis and tested positive for *Mycobacterium microti*. (a) Overview of a granulomatous lymphadenitis showing focal‐extensive necrotic cores and dystrophic calcifications (arrow heads). The necrotic cores are surrounded by a mixed population of inflammatory cells and by a wide capsule of connective tissue (arrows), haematoxylin and eosin (HE; 40×). (b) Scanty acid‐fast rods are visible in the necrotic debris. Occasionally, S‐shaped bacilli, which are commonly associated with *Mycobacterium microti* were observed intra‐ and extracellularly, Ziehl‐Neelsen stain (40×). (c) Granuloma characterized by focal‐extensive necrosis and mild inflammatory infiltration of epithelioid macrophages, neutrophils, multinucleated Langhans type giant cells (arrows) and eosinophils is shown (HE; 40×)

Of the lymph nodes from the 101 wild boars showing growth of *Mycobacterium* spp., 25.7% showed macroscopic pathological lesions (Table 2). Macroscopic visible lesions such as single or multiple caseous, necrotic and calcified nodules of different sizes (1–30 mm) were observed (*n* = 14). In addition to the described nodular lesions, lymph node enlargement, discoloration and induration were recorded (*n* = 26). Histologically, in 70% of the samples presenting nodular lesions, a moderate granulomatous lymphadenitis with scarce giant cells of Langhans type surrounding or adjacent of a mild to moderate focal‐extensive necrosis was visible. Additionally, the lymph nodes showed a moderate to severe reactive hyperplasia and a mild to moderate eosinophilic lymphadenitis. *Mycobacterium avium* subsp. *hominissuis*, the predominant species identified in the present study, was found in lymph nodes with and without macroscopic changes. On the contrary, *M. microti* (*n* = 5) and *M. florentinum* (*n* = 3) infections were always associated with visible lesions such as caseous, necrotic and calcified nodules. A subset of lymph nodes (*n* = 14) presenting macroscopic lesions compatible with mycobacterial infections were examined histologically. Lymph nodes of animals infected with *M. microti* and *M. florentinum* showed all granulomatous lymphadenitis characterized by focal‐extensive necrosis and mild inflammatory infiltration of epithelioid macrophages, neutrophils, multinucleated Langhans giant cells and eosinophils (Figure 3a,c). In three cases, dystrophic calcifications were markedly present. Overall, the lesions observed macroscopically and histologically were circumscribed and of mild to moderate entity. Ziehl‐Neelsen staining revealed scanty AFBs according to the International Union against Tuberculosis and Lung Disease (IUATLD) and WHO grading scales. Samples that presented visible lesions compatible with tuberculosis and tested positive for *M. microti* showed ''croissant‐like'' or S‐shaped AFBs, which is commonly associated with this species (van Soolingen et al., 1998). Acid‐fast bacilli were observed extracellular and within macrophages (Figure 3b).

**TABLE 2:** Macroscopic and histological findings compatible with mycobacterial infection in lymph nodes of hunted wild boars positive for *Mycobacterium* spp. are resumed

Examination of lymph nodes			Species of mycobacteria identified
*n*	Macroscopic findings^a^	Histological findings^b^	
10	Single or multiple nodules	+	*Mycobacterium microti* (4) *Co‐infection M. microti* and *Mycobacterium neoaurum* (1) *Mycobacterium florentinum* (3) *Mycobacterium avium* subsp. *hominissuis* (1) *Mycobacterium nonchromogenicum* (1)
4	Single or multiple nodules	–	*M. avium* subsp. *hominissuis* (1) *Mycobacterium vulneris* (1) *Mycobacterium lentiflavum* (1) *Mycobacterium neoaurum* (1)
12	+	nd	*M. avium* subsp. *hominissuis* (9) *M. nonchromogenicum* (3)
75	–	nd	All species listed in Table 1 with the exception of *M. microti* and *M. florentinum*

Note

+, macroscopic or histological finding present; – no macroscopic or histological findings; nd, not done.

^a^ Macroscopic findings comprised in all cases enlargement, discoloration and induration.

^b^ Histological findings comprised focal‐extensive necrosis (mild to moderate), granulomatous lymphadenitis (moderate) as well as reactive hyperplasia (moderate to severe) and eosinophilic lymphadenitis (mild to moderate).

### 3.2 MALDI‐TOF mass spectrometry

By setting a LSV cut‐off at ≥2.0, MALDI‐TOF MS was able to correctly identify 80 isolates out of 111 (72.1%) appertaining to 19 species according to sequence analysis (16s rRNA gene, *rpoB* and *hsp65*). By lowering the cut‐off for species identification at ≥1.8 as previously proposed for mycobacterial species (Alcaide et al., 2018; Pranada, 2015), 100 isolates (90.1%) from the same 19 species were correctly assigned (Table 1). Two isolates, one *M. vulneris* misidentified as *M. colombiense* (LSV 2.08) and a second one, presumably representing a new species and misidentified as *M. arupense* (LSV 1.81) led to discrepant results (Supplementary Material S2–S4). The remaining 9 isolates, classified as 'no identification possible' by MALDI‐TOF MS, were identified by sequence analysis as *M. colombiense* (3/3 isolates) *M. scrofulaceum* (2/2 isolates), *M. monacense* and *M. terrae* complex (one isolate each), and two additional undescribed *Mycobacterium* species.

## 4 DISCUSSION

### 4.1 *Mycobacterium tuberculosis* complex

In five out of 176 analysed wild boars (2.8%; CI_95%_ 1.2–6.5), DNA appertaining to a member of the MTBC was detected by qPCR. The observed prevalence is in accordance with a previous study in wild boars originating from three different Swiss Cantons, where the observed prevalence was 3.6% (Schoning et al., 2013). Spoligotyping (SB0118) and molecular characterization based on MIRU‐VNTR revealed a common source of infection or a transmission chain of *M. microti* (Supplementary Material S1). *Mycobacterium microti* has been described to cause extensive lesions indistinguishable from those caused by other MTBC members in wild boars and immunocompetent humans. Certain strains require prolonged incubation time or are unculturable on traditional solid and liquid media. Because of the very poor sensitivity of culture, a molecular approach is crucial for the detection of *M. microti* (Boniotti et al., 2014; Emmanuel et al., 2007; Frank, Reisinger, Brandt‐Hamerla, Schwede, & Handrick, 2009; Geiss, Feldhues, Niemann, Nolte, & Rieker, 2005; Niemann et al., 2000; van Soolingen et al., 1998).

Identical spoligotype and MIRU‐VNTR profiles (ETRs loci) were observed by Boniotti and colleagues in five out of 26 isolates from Italian wild boars analysed between 2007 and 2009 (Boniotti et al., 2014). Over this period, a slightly higher prevalence of 5.8% was described in Italy. This suggests a persistent presence of one *M. microti* strain affecting the local wild boar population over at least a decade. Based on these findings, a transboundary presence of *M. microti* in the Swiss and the Italian border region along the Province of Como is shown. To date, spoligotype profile SB0118 is the only *M. microti* profile detected in Switzerland and it was isolated from a large host range including domestic cats, South American camelids and captive gibbons (unpublished results). Interestingly, different wild rodent species originating from hotspot areas examined for MTBC were all tested negative by molecular and cultural methods (manuscript in preparation).

### 4.2 *Mycobacterium avium* complex

The predominant species identified in this study belonged to the MAC (Table 1). This complex comprises several clinically important mycobacterial species (van Ingen, Turenne, Tortoli, Wallace, & Brown‐Elliott, 2018). *Mycobacterium avium* (*n* = 25) was the most prevalent member of the complex, followed by *M. colombiense* (*n* = 3), *M. chimaera/intracellulare* group (*n* = 2) and *M. vulneris* (*n* = 1; Table 1). *M. avium* is a thermophilic slowly growing *Mycobacterium* and comprises four subspecies, namely *M. avium* subsp. *avium* (*Maa*)*, M. avium* subsp. *silvaticum* (*Mas*), *M. avium* subsp. *hominissuis* (*Mah*) and *M. avium* subsp. *paratuberculosis* (*Map*). Sequencing of the 3′ region of the *hsp65* gene can unambiguously distinguish between these subspecies (Turenne et al., 2006). *M. avium* subsp. *hominissuis* has the broadest host range compared to the other members of the MAC; nevertheless, a clear differentiation between environmental and host‐specific members of the MAC is necessary to better understand its distribution, host‐adaptation and clinical implications (Turenne et al., 2006). Because NTM infections are not notifiable to public health authorities in most countries, data regarding the incidence and prevalence of diseases caused by these agents are lacking. Identification of potential infection sources however, is of great relevance. Among the two species of NTM that have been most frequently isolated in the present study (*M. avium* and *M. nonchromogenicum*), MAC members have been described as the most common cause of mycobacteriosis in human in Northern Europe (Hoefsloot et al., 2013), Japan (Nishiuchi, Iwamoto, & Maruyama, 2017), Korea (Ko et al., 2018) and North America (Boyle, Zembower, Reddy, & Qi, 2015). Recently, possible MAC reservoirs and infection sources for humans and animals including drinking water, bathrooms and hot tubs have been investigated using molecular analyses (Eisenberg et al., 2012; Falkinham, Iseman, de Haas, & van Soolingen, 2008; Hilborn et al., 2008). Noteworthy, a close genetic relatedness between human and swine isolates has been reported (Johansen et al., 2007; Mobius et al., 2006).

### 4.3 Prevalence of Mycobacteria in relation to age

Adult wild boars were more prone to be infected with mycobacteria in comparison with juvenile animals or yearlings (Figure 2a), suggesting that the isolated mycobacteria are probably the result of a prolonged infection or colonization more than a transient presence. This is corroborated by the observation that co‐infections, including one animal infected with *M. microti*, were mostly in adult animals and none of the wild boars from the juvenile group was infected with multiple species. Contrary to the observations from a Spanish study (Garcia‐Jimenez et al., 2015) where MAC was isolated more often from subadults, no statistically significant correlation between MAC and the age of the animals was observed in the present survey (Figure 2b). Co‐infections with MTBC/NTM or two different NTM have been described in animals and humans (Garcia‐Jimenez et al., 2015; Gcebe & Hlokwe, 2017; Lim et al., 2011; Stepanyan et al., 2019; Yilmaz, Ucar, & Saglam, 2017). A possible explanation for this increased number of co‐infection in older individuals may be a cumulative effect with time and exposure. Overall, no effect of NTM isolation and MTBC infection could be observed due to the small number of MTBC‐positive animals and one animal being co‐infected with *M. microti* and *M. neoaurum*.

### 4.4 Wild boar and Mycobacteria

Wild boars (*Sus scrofa*) are among the most widely distributed large mammals worldwide. Their natural range extends from Western Europe and the Mediterranean basin to Eastern Russia, Japan and South‐East Asia (Massei et al., 2015). The high mobility associated with the highest reproductive capacity among ungulates enable an annual population growth rate of 250% under favourable food and weather conditions (Ebert, Knauer, Spielberger, Thiele, & Hohmann, 2012; Gethoffer, Sodeikat, & Pohlmeyer, 2007). Supplemental feeding of wild ungulates is prohibited by law in several Swiss Cantons, including Ticino. Despite this order and the annual population reduction by hunters, the population in the study area is rising, augmenting the animal‐to‐animal as well as the animal‐to‐human contact probabilities.

Since the hunted animals of the present study were judged to be in an overall healthy condition and suitable for human consumption, the *Mah* infection rate of 14% observed in the mandibular lymph node is noteworthy and represents a veterinary public health concern. Mandibular lymph nodes have been described to be the preferred entry point for mycobacteria in wild boars (Queiros et al., 2019). This is probably due to their eating habits allowing the intake of environmental contaminations and infected feed sources. After oronasal infection, viable microorganisms often concentrate in mandibular lymph nodes and from there are eliminated, persist or eventually disseminate throughout other organ systems. Mandibular lymph nodes are the most likely organ for visible lesion caused by MTBC in adult animals and in a relevant proportion of cases this is the only organ affected (Dondo et al., 2007; Martin‐Hernando et al., 2007; Munoz‐Mendoza et al., 2013).

Only few publications extensively investigated the presence of NTM in wild boar (Garcia‐Jimenez et al., 2015; Gortazar et al., 2011; Pate et al., 2016; Trcka et al., 2006). *M. chelonae* was the most frequent NTM species isolated by García‐Jiménez *et al*., while Gortazar and colleagues did not detect *M. chelonae* in 124 wild boars tested. In the present study, *M. chelonae* was not detected, suggesting a diversity of NTM species at geographical level. Members of the MAC were also irregularly detected and some of them, e.g., *M. intracellulare*, *Maa*, *Mah* and *M. colombiense* are recognized as opportunistic pathogens in human and veterinary medicine (Table 1). It is therefore fundamental to implement advanced approaches in order to identify the exact species involved and estimate their relevance as potential infection source for consumers.

Previous reports described NTM isolation rates in wild pigs varying from 8.9% (Trcka et al., 2006), 16.1% (Gortazar et al., 2011), 16.8% (Garcia‐Jimenez et al., 2015) to 18.2% (Pate et al., 2016). In addition to the above‐mentioned NTM, *M. peregrinum*, *M. nebraskense*, *M. lentiflavum*, *M. nonchromogenicum*, *M. engbaeki* and *M. septicum* were isolated from wild boar in recent studies from Brazil (Lara et al., 2011), Czech Republic (Trcka et al., 2006), Italy (Boniotti et al., 2014), Slovenia (Pate et al., 2016) and Spain (Garcia‐Jimenez et al., 2015; Gortazar et al., 2011). Among the remaining species isolated in the present study for the first time, *M. florentinum* is noteworthy regarding the granulomatous lesions observed in the three affected animals.

In contrast to packs, which tend to use only a small portion of their territory and move to another range at regular intervals, the adult wild boar male has a single territory that can range up to 50 km^2^. These individuals are able to move across the entire territory at daily basis, playing a crucial role in the spread of animal and zoonotic pathogens (Nugent, Gortazar, & Knowles, 2015; Schulz et al., 2019; Spitz, 1992). This high mobility may also be one of the possible explanations for the homogenous spread of the NTM species isolated across Canton of Ticino.

Because of their rooting behaviour and eating habits, wild boars are often in contact with environmental NTM species. Moreover, as for badgers, the occasional consumption of dead small rodents and other carrion can enable direct transmission of pathogenic mycobacteria and, eventually, contamination of pasture, water and cattle feed (Munoz‐Mendoza et al., 2013). However, this does not seem to cause generalization and clinical disease in all individuals, presumably because of the previously suggested genetic resistance of wild boars against bTB causing agents and possibly other mycobacterial species (Acevedo‐Whitehouse et al., 2005; Dondo et al., 2007). Nevertheless, in the present study, macroscopic lesions suspicious or undistinguishable from bTB changes were observed in 44% *Mah*‐infected wild boars, in accordance with previously published data (Munoz‐Mendoza et al., 2013).

### 4.5 Macroscopic and histological findings

An interesting finding was the presence of granulomatous lesions compatible with tuberculosis observed in a subset of lymph nodes analysed. In particular, samples in which *M. microti* and *M. florentinum* were detected, showed granulomatous lymphadenitis characterized by focal‐extensive necrosis and in some cases dystrophic calcifications. These calcifications and the paucibacillary nature of the lesions suggest, however, a circumscribed and chronic process. Because of the design of the present study, it was not possible to assess the presence of further lesions in the wild boar carcasses. To the authors’ knowledge, this is the first time that similar lesions caused by *M. florentinum* are described in veterinary medicine. Classified as a slowly growing *Mycobacterium*, *M. florentinum* is an opportunistic pathogen isolated from immunocompetent and immunocompromised humans with pulmonary disorders and lymphadenitis (Tortoli et al., 2005). Macroscopically and histologically the lesions caused by *M. microti* and *M. florentinum* in wild boars were indistinguishable. These findings highlight the importance of a molecular approach that allows a rapid and reliable differentiation of MTBC members from other NTM. The geographical distribution of *M. florentinum* appears to be widespread since human cases have been described in Italy, Finland, Japan and the United States (Nukui et al., 2014; Syed, Aderinboye, Hanson, & Spitzer, 2010; Tortoli et al., 2005).

A relevant number of *Mycobacterium* spp. other than *M. microti* and *M. florentinum* were isolated from tissue samples with no lesions (Table 2). Similar findings were recently described by two studies focused on lymph nodes of slaughter pigs (Mann, Dzieciol, Metzler‐Zebeli, Wagner, & Schmitz‐Esser, 2014; Muwonge et al., 2012). The presence of viable mycobacteria without evidence of histopathological granulomatous lesions might represent an early stage of the infection, which is not yet morphologically identifiable and in most cases probably results in the elimination of the microorganism (Mann et al., 2014). Contamination with environmental mycobacteria is not to be excluded. Because of the small size of the samples, disinfection of the mandibular lymph nodes superficial area was not possible. Based on the high sensitivity of the MTBC‐specific qPCR adopted, cross‐contamination of the samples can be excluded. *M. microti* DNA was identified exclusively in lymph nodes presenting visible lesions and from animals processed on different days.

### 4.6 Identification of Mycobacteria with MALDI‐TOF mass spectrometry

Overall, MALDI‐TOF MS showed to be reliable for the identification of NTM derived from veterinary specimens. Although the threshold recommended by the manufacturer enabled the correct identification of only 72.1% of the isolates, a lower cut‐off for mycobacteria is commonly used (Alcolea‐Medina et al., 2019; Buchan, Riebe, Timke, Kostrzewa, & Ledeboer, 2014; Mediavilla‐Gradolph et al., 2015; Saleeb et al., 2011). In the authors’ opinion, an important drawback of the MALDI‐TOF MS technology in comparison with sequence analysis of housekeeping genes such as 16S rRNA, is that yet undescribed species will go undetected, either without any identification or as misidentification (S2–S4). In this study, three NTM species: *M. colombiense* (3/3 isolates), *M. scrofulaceum* (2/2 isolates) and *M. monacense* (one isolate) could not be identified although present in the MBT Mycobacteria Library 4.0. The most probable explanation for this is the restricted number of MSP (main spectrum profiles) present in the library used as reference. Moreover, one isolate of *M. vulneris* was misidentified as *M. colombiense* with a LSV of 2.08, demonstrating that closely related species still represent a challenge to be unambiguously identified and require sequence analysis for accurate assignment. The discrimination of *Map* with the MALDI‐TOF MS technique has been assessed in previous studies (Ravva, Harden, & Sarreal, 2017; Ricchi et al., 2017). The 25 *M. avium* isolates tested in the present study were all confirmed to be *Mah* by sequence analysis, and the first subspecies suggestion provided by MALDI‐TOF MS was generally correct (80%). However, differentiation of *M. avium* subspecies with MALDI‐TOF MS has to be performed with carefulness and might require additional gene sequence analysis.

Three *M. diernhoferi* isolates were misidentified by BLAST analysis of the *rpoB* gene sequences. According to NCBI BLAST, the closest species was *M. aurum* with a percentage identity score of 97%. The MALDI‐TOF MS analysis suggested *M. diernhoferi* with an unequivocal LSV ≥ 2.0 for all three strains. Hence, a NCBI nucleotide search for *M. diernhoferi rpoB* sequences allowed the finding of an identical sequence, namely whole genome shotgun sequence derived from strain IP141170001 BioProject PRJNA354248. In our experience, this is a rare case, where only whole genome sequences are available for a specific species in the database and these are not included for similarity search.

## 5 CONCLUSION

In conclusion, a remarkable number of mandibular lymph nodes collected from wild boars presented viable mycobacteria. Although the zoonotic risk of the isolated NTM remains unclear, it must be emphasized that the hunted animals were intended for human consumption and among the isolated species (*n* = 24), the large majority (*n* = 18) has been described as human pathogens. In addition, the present findings show that MALDI‐TOF MS has a high concordance rate to the reference method and because of his rapidness, cost‐effectiveness and high throughput represent a valid diagnostic tool for identification of NTM species in veterinary medicine.

## CONFLICT OF INTEREST

The authors declare that they have no conflict of interest.

## Supporting information

Supinfo S1

Supinfo S2‐S4

## Data Availability

The data that support the findings of this study are available from the corresponding author upon reasonable request.
