# Supplementary material for: Mycobacterial infections in wild boars (Sus scrofa) from Southern Switzerland: Diagnostic improvements, epidemiological situation and zoonotic potential
Source: Transbound Emerg Dis. 2020 Jul 20;68(2):573–86. doi: 10.1111/tbed.13717 (PMC8247353; doi:10.1111/tbed.13717)
Supplement: Supplementary file 1 — Supinfo S1 [file TBED-68-573-s001.docx]

# Mycobacterial Infections in Wild Boars (*Sus scrofa*) from Southern Switzerland: Diagnostic Improvements, Epidemiological Situation and Zoonotic Potential

# Giovanni Ghielmetti, Monika Hilbe, Ute Friedel, Chiara Menegatti, Luca Bacciarini, Roger Stephan, Guido Bloemberg

**Supplementary material S1. Common MIRU-VNTR allele profile of four out of five *M. microti* containing samples.** The MIRU-VNTR code, suggestive for a common source of infection or a transmission chain is compared with the reference strain *M. tuberculosis* H37Rv in the 24 MIRU-VNTR standard panel.

| ***M. tuberculosis* H37Rv** | 2 | 2 | 4 | 3' | 1 | 3 | 2 | 2 | 2 | 5 | 3 | 4 | 2 | 3 | 6 | 1 | 3 | 3 | 3 | 3 | 5 | 5 | 2 | 2 | 2 | 3 |
| --- | --- | --- | --- | --- | --- | --- | --- | --- | --- | --- | --- | --- | --- | --- | --- | --- | --- | --- | --- | --- | --- | --- | --- | --- | --- | --- |
| ***M.*** ***microti from Swiss wild boars*** | **2** | **3** | **4** | **4** | **2** | **4** | **4** | **3** | **1** | **6** | **9** | **3** | **4** | **3** | **4** | **2** | **2** | **2** | **3** | **1** | **3** | **9** | **3** | **2** | **2** | **3** |
