# Supplementary material for: Mycobacterial infections in wild boars (Sus scrofa) from Southern Switzerland: Diagnostic improvements, epidemiological situation and zoonotic potential
Source: Transbound Emerg Dis. 2020 Jul 20;68(2):573–86. doi: 10.1111/tbed.13717 (PMC8247353; doi:10.1111/tbed.13717)
Supplement: Supplementary file 2 — Supinfo S2‐S4 [file TBED-68-573-s002.docx]

# Mycobacterial Infections in Wild Boars (*Sus scrofa*) from Southern Switzerland: Diagnostic Improvements, Epidemiological Situation and Zoonotic Potential

# Giovanni Ghielmetti, Monika Hilbe, Ute Friedel, Chiara Menegatti, Luca Bacciarini, Roger Stephan, Guido Bloemberg

**Supplementary material S2.** Alignment of the 16S rRNA gene sequences of *Mycobacterium arupense* ATCC BAA-1242^T^ reference strain (GenBank accession no. DQ157760.2) and isolate 17-1543 which was misidentified as *M. arupense* by matrix-assisted laser desorption ionization–time of flight (MALDI-TOF) mass spectrometry*.* Isolate 17-1543 shows two SNPs and two deletions out of 1144 bp of the 16S ribosomal RNA gene.


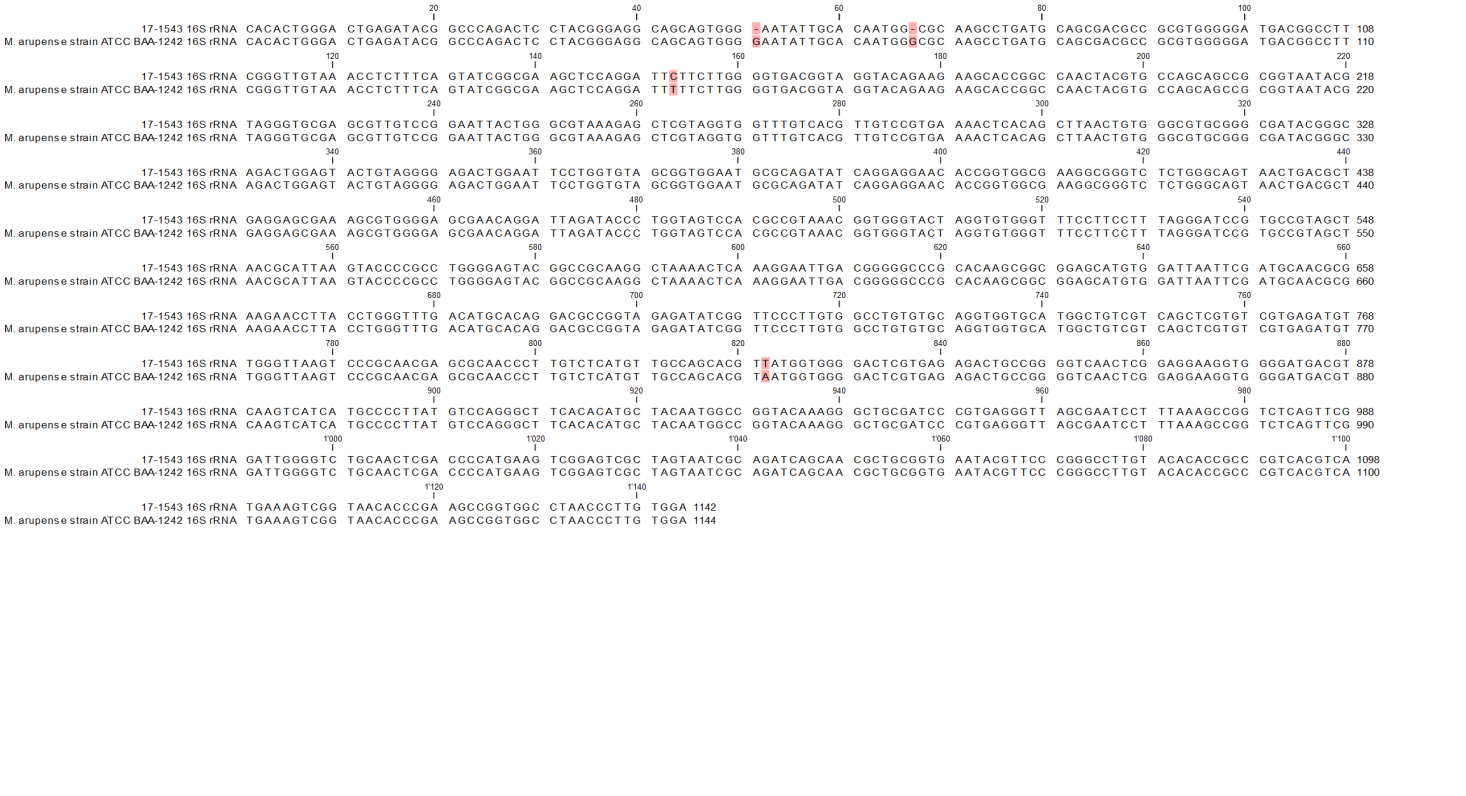


**Supplementary material S3.** Alignment of the *rpoB* gene sequences of *Mycobacterium arupense* ATCC BAA-1242^T^ reference strain (GenBank accession no. KT861786.1) and isolate 17-1543 which was misidentified as *M. arupense* by matrix-assisted laser desorption ionization–time of flight (MALDI-TOF) mass spectrometry*.* Isolate 17-1543 shows 36 SNPs out of 735 bp of the *rpoB* gene. The resulting identity score of 95% is below the threshold proposed for this gene by Adékambi and colleagues for intra-species variabilities ^1^. These findings indicate that isolate 17-1543 should not to be classified as *Mycobacterium arupense*.


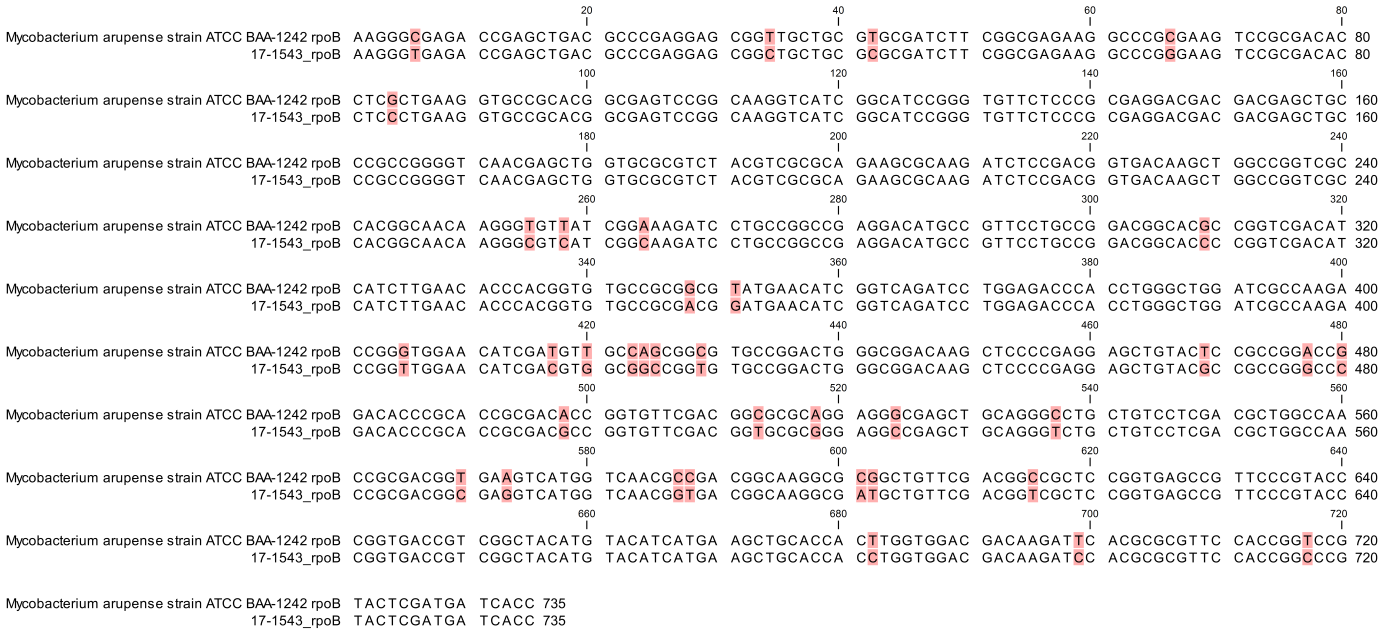


**Supplementary material S4.** Alignment of the *hsp*65 gene sequences of *Mycobacterium arupense* strain ATCC BAA-1242^T^ reference strain (GenBank accession no. JF491325.1) and isolate 17-1543 which was misidentified as *M. arupense* by matrix-assisted laser desorption ionization–time of flight (MALDI-TOF) mass spectrometry. Isolate 17-1543 shows 13 SNPs out of 400 bp of the *hsp*65 gene with a resulting identity score of 96.7%.


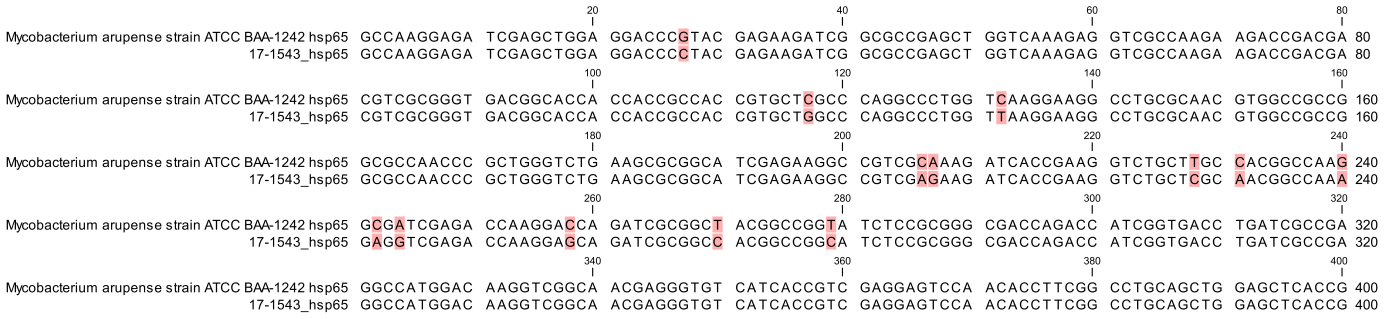


1 Adékambi, T., Colson, P. & Drancourt, M. rpoB-based identification of nonpigmented and late-pigmenting rapidly growing mycobacteria. *J. Clin. Microbiol.* **41**, 5699-5708 (2003).
